# Glucose-Reducing Effect of the ORMD-0801 Oral Insulin Preparation in Patients with Uncontrolled Type 1 Diabetes: A Pilot Study

**DOI:** 10.1371/journal.pone.0059524

**Published:** 2013-04-09

**Authors:** Roy Eldor, Ehud Arbit, Asher Corcos, Miriam Kidron

**Affiliations:** 1 Diabetes Unit, Internal Medicine, Hadassah University Hospital, Jerusalem, Israel; 2 Oramed Pharmaceuticals, Jerusalem, Israel; 3 Diabetes and Endocrine Clinic, General Health Services, Affiliated to Hadassah Medical School, Jerusalem, Israel; University of Texas Health Science Center at San Antonio, United States of America

## Abstract

The unpredictable behavior of uncontrolled type 1 diabetes often involves frequent swings in blood glucose levels that impact maintenance of a daily routine. An intensified insulin regimen is often unsuccessful, while other therapeutic options, such as amylin analog injections, use of continuous glucose sensors, and islet or pancreas transplantation are of limited clinical use. In efforts to provide patients with a more compliable treatment method, Oramed Pharmaceuticals tested the capacity of its oral insulin capsule (ORMD-0801, 8 mg insulin) in addressing this resistant clinical state. Eight Type I diabetes patients with uncontrolled diabetes (HbA1c: 7.5–10%) were monitored throughout the 15-day study period by means of a blind continuous glucose monitoring device. Baseline patient blood glucose behavior was monitored and recorded over a five-day pretreatment screening period. During the ensuing ten-day treatment phase, patients were asked to conduct themselves as usual and to self-administer an oral insulin capsule three times daily, just prior to meal intake. CGM data sufficient for pharmacodynamics analyses were obtained from 6 of the 8 subjects. Treatment with ORMD-0801 was associated with a significant 24.4% reduction in the frequencies of glucose readings >200 mg/dL (60.1±7.9% pretreatment vs. 45.4±4.9% during ORMD-0801 treatment; *p* = 0.023) and a significant mean 16.6% decrease in glucose area under the curve (AUC) (66055±5547 mg/dL/24 hours vs. 55060±3068 mg/dL/24 hours, *p* = 0.023), with a greater decrease during the early evening hours. In conclusion, ORMD-0801 oral insulin capsules in conjunction with subcutaneous insulin injections, well tolerated and effectively reduced glycemia throughout the day.

**Trial Registration:**

Clinicaltrials.gov NCT00867594.

## Introduction

Type 1 diabetes mellitus (T1DM) is commonly characterized by instability and unpredictability of glycemic readings, resulting in poor glycemic control. While the underlying causes can be diverse [1], uncontrolled hepatic gluconeogenesis is suggested to play a central role [2].

As the major organ capable of producing and assimilating substantial amounts of glucose, and responsible for maintaining blood glucose concentrations within a tight range, the liver poses a prime target for insulin delivery. In the postprandial state, portal insulin concentrations are critical for sequestering approximately one third [3] of the glucose load and for suppressing basal glucose production by 65–80% [4], [5]. In the fasting state, portal insulin tightly regulates hepatic glucose production, effectively modulating hepatic glycogenolysis. Moreover, the liver is exquisitely sensitive to changes in portal insulin concentrations as evident from studies in dogs [6] and in healthy subjects [7], [8], where even small increases effectively inhibited glycogenolysis.

Restoration of normal portal insulin/glucagon ratios may enable tighter regulation of gluconeogenesis and glycogenolysis [9], however, subcutaneously administered insulin is sometimes inadequate in inducing such a state. Direct intra-peritoneal insulin delivery systems have led to improved glycemic control and to a smaller number of hypoglycemic events among uncontrolled T1DM patients [10]. Orally administered insulin is speculated to induce similar effects, while offering the benefit of hepatic first-pass insulin metabolism, reduced systemic exposure and ease-of-use [11].

Here, we describe a pilot study, whereupon ORMD-0801, a newly developed oral insulin preparation, was administered to uncontrolled T1DM patients, alongside their daily insulin regime. The study assessed ORMD-0801 tolerability and the impact of the orally delivered insulin in combination with standard patient insulin therapy, on the stability of glycemic readings.

## Methods

The protocol for this trial and supporting CONSORT checklist are available as supporting information; see Checklist S1 and Protocol S1.

### Ethics statement

The study was approved by the institutional review board of the Hadassah University Hospital.

### Inclusion criteria and patient demographics

Male or female subjects with poorly controlled T1DM (HbA1c: 7.5–11%) regularly treated with multiple daily insulin analog injections or continuous subcutaneous insulin analog injection, and otherwise healthy, were included in this study. T1DM had been diagnosed earlier by each patient's physician, by means of clinical characteristics, namely, complete insulin dependence and brittle glucose profiles, as well as by positive glutamic acid decarboxylase (GAD) antibodies tests. Patient demographics and baseline diabetes-related profiles are summarized in Table 1.

**Table 1:** Patient demographics and baseline characteristics.

	Mean (Range)
Age (years)	37 (27–50)
Gender	6 male; 2 female
Diabetes Duration (years)	16 (10–26)
HbA1c (%)	9.4 (7.56–11.04)
Diabetes-related complications	1/8 CVA- recovered
	1/8 Retinopathy- controlled
Concomitant diseases	2/8 Dyslipidemia-controlled
	2/8 Depression-controlled
	2/8 Hypertension-controlled
	1/8 Hyperthyroidism- controlled

### Study design

**Figure 1:**
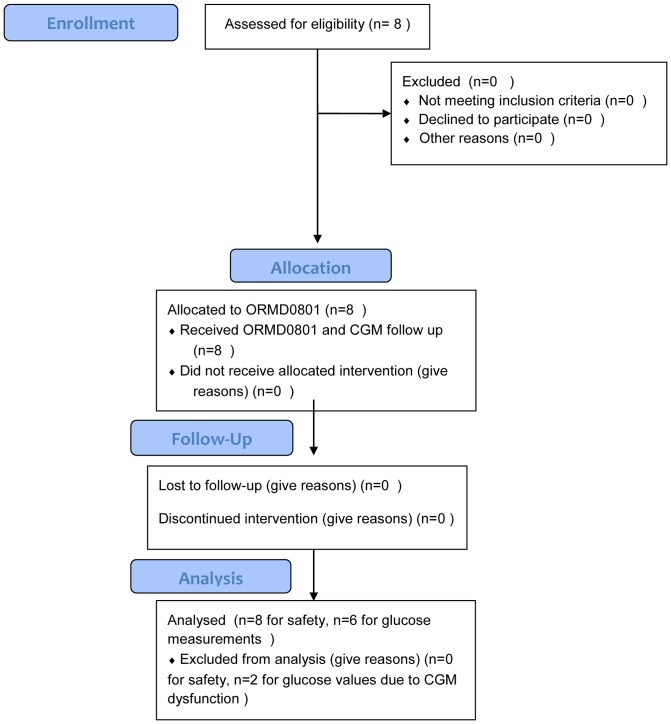
Study flow diagram.

This was an open-label, single center study of a specific formulation of ORMD-0801 capsules, each containing 8 mg insulin, administered to 8 Type I diabetes patients three times daily. Following signed, informed consent and a complete physical and laboratory evaluation, patients were monitored over a 15-day study period by means of a continuous glucose monitoring device (CGM, Medtronic MiniMed, Northridge, CA, USA) (Figure 1). Baseline patient blood glucose behavior was determined over the initial five-day pretreatment screening period, which was scheduled within 14 days of the start of the treatment period. In the ensuing ten-day treatment phase, patients were asked to eat and continue diabetes treatment regimens as usual and to self-administer the oral insulin capsule (ORMD-0801) three times daily, 45 min prior to meals. Patients were blinded to the CGM recordings throughout the study. The average five-day CGM sensor lifetime required sensor replacement three times throughout the monitoring period. A trained CGM technician and the trial nurse were responsible for replacing the sensor.

All patients were on a stable insulin dose for at least 6 months prior to the study. Moreover, the patients were instructed not to change their insulin doses or glucose measurement regimen during the study period. At the end of the study patients were specifically asked about changes in the insulin dose during the study and none were noted. The patients were blinded to the CGM data throughout the study.

### Analysis

Analysis of adverse events data was performed on all 8 subjects. Parameters, analyzed for change from baseline values included vital signs, laboratory evaluations, physical examination findings and patient self-reporting of adverse events throughout the trial. Furthermore, frequency of glucose recordings in the hypoglycemic range (below 70 mg/dL) was analyzed. Glycemic data sufficient for analysis was obtained from 6 of the 8 subjects. Mean area under the curve for glucose, %time below 70 mg/dL and %time over 200 mg/dL were compared with the single sided non-parametric Wilcoxon signed ranks test using IBM SPSS statistics software, version 20.

## Results

**Figure 2:**
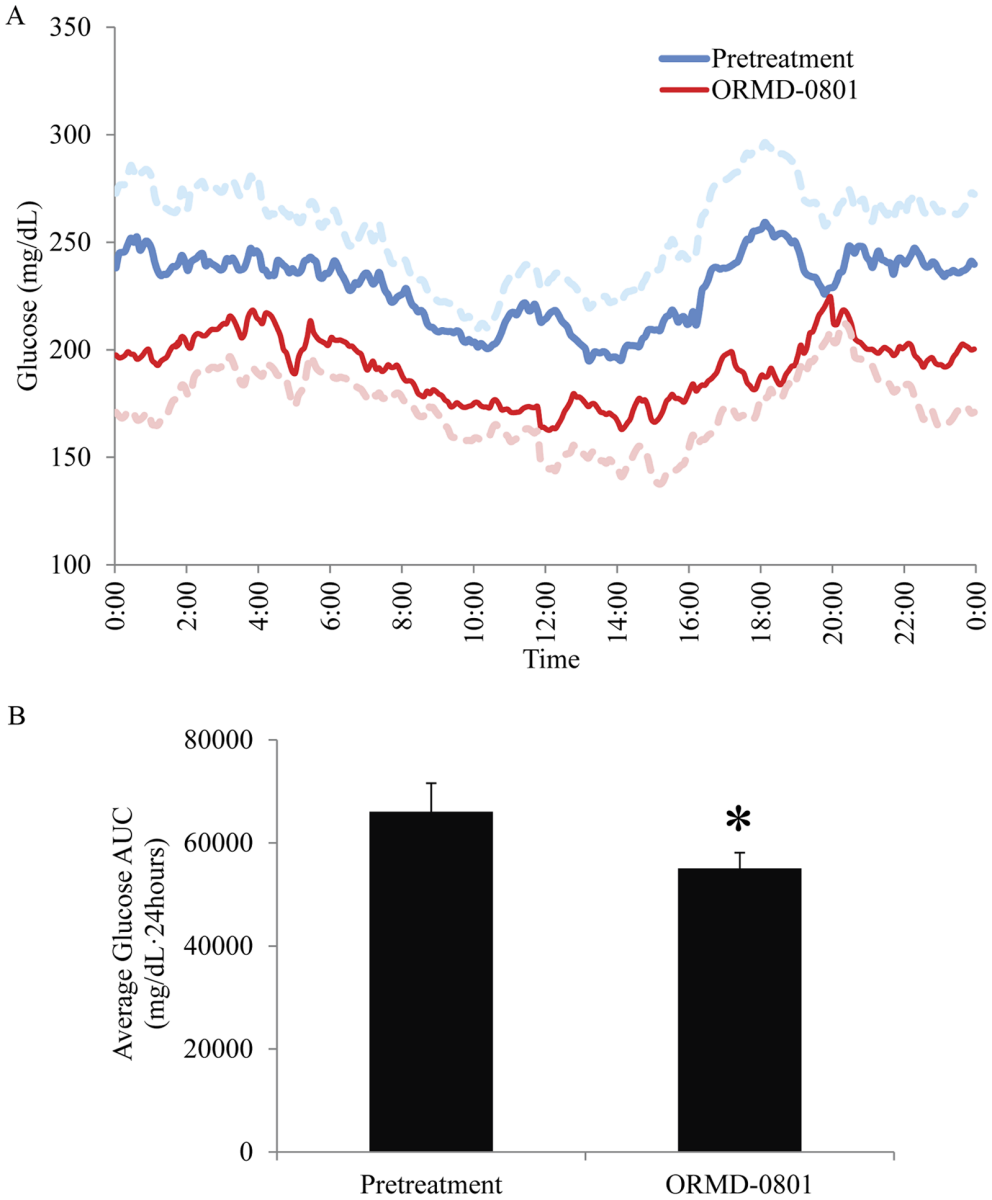
Mean glucose concentrations before and during ORMD-0801 oral insulin support therapy. A. Mean blood glucose levels of six Type I diabetic subjects, continuously monitored throughout the pretreatment (blue) and ORMD-0801, oral insulin-treatment (red) phases (dotted lines represent the corresponding standard errors). The greatest reduction (21.2%) is noted between 5–7pm. B. ORMD-0801 treatment was associated with a mean 16.6% decrease in glucose area under the curve (AUC) (66055±5547 mg/dL/24 hours before treatment vs. 55060±3068 mg/dL/24 hours during ORMD-0801 treatment,*- *p* = 0.023).

In addition to full compliance with the study protocol, patients reported full adherence to their regular insulin therapy regimen during the study period. No adverse events or hypoglycemic attacks were reported throughout the study by any of the eight patients. All liver function tests performed during and after the study indicated normal functioning. Biochemical hypoglycemia analyses revealed a tendency towards more readings below 70 mg/dL during ORMD-0801 treatment, with an average 0.45%±0.2 of readings <70 mg/dL in the pretreatment phase, versus 1.99%±0.88 of readings while on ORMD-0801 support (Table 2; p = 0.069). Due to the small sample size it is impossible to predict whether this tendency is of clinical relevance and will become significant in a larger cohort followed for a longer time. In parallel, a 24.4% reduction in the frequencies of glucose readings >200 mg/dL was observed during ORMD-0801 therapy (Table 2; 60.1±7.9% pretreatment vs. 45.4±4.9% during ORMD-0801 treatment; *p* = 0.023). CGM data sufficient for pharmacodynamics analyses were obtained from 6 of the 8 subjects. ORMD-0801 treatment resulted in a significant mean 16.6% decrease in glucose area under the curve (AUC) (66055±5547 mg/dL/24 hours before treatment vs. 55060±3068 mg/dL/24 hours during ORMD-0801 treatment, *p* = 0.023),, with the greatest reduction (21.2%) between 5–7pm (Figure 2).

**Table 2:** Frequency of recordings of blood glucose concentrations below 70 mg/dL or above 200 mg/dL (% of total measurements obtained by continuous glucose monitoring).

% Measurements<70 mg/dL			% Measurements>200 mg/dL	
	Pretreatment	ORMD-0801	Pretreatment	ORMD-0801
pt1	0	0	82	69.1
pt2	0	3.87	68.5	38.29
pt3	0.99	0	28.6	38.49
pt4	1.04	4.31	57.2	40.9
pt5	0	0.07	75	47.78
pt6	0.69	3.69	49.3	37.8
Mean	0.45	1.99	60.1	45.39
se	±0.21	±0.88	±7.94	±4.98
*t-test*	p = 0.069		p = 0.023	

In short, addition of pre-prandial ORMD-0801 oral insulin treatment was well tolerated and led to a statistically and clinically significant reduction in glycemia in this small cohort of patients.

## Discussion

Due to the diversity of diabetes etiology and the multiplicity of factors exacerbating its course, combination therapies are often necessary to achieve clinically satisfactory results. The present study demonstrates a real-life application of insulin-based capsules in the regulation of poorly controlled T1DM. Apart from being clinically well tolerated, the addition of ORMD-0801 oral insulin capsules to subcutaneous insulin injection regimens among type I diabetes patients resulted in a synergistic reduction in blood glucose concentrations which was most prominent during the early evening hours. This was accompanied by a tendency towards more biochemical hypoglycemia readings. The clinical relevance of this is unclear and may reach statistical significance in a larger and more prolonged trial.

This study was not designed to address the question of whether the hypoglycemic action of the addition of ORMD-0801 to the subcutaneous insulin regime, results from a first-pass hepatic effect leading to reduced post-prandial hepatic glucose production or a cumulative peripheral effect of both s.c. and oral insulin on peripheral glucose uptake. Moreover, the small sample size, study design and short follow up period prevent us from reaching strict conclusions and can only be addressed as hypothesis forming for future larger and longer trials planned. Nonetheless, the observed 16.6% reduction in glycemia will prove clinically relevant if shown to translate to a similar reduction in both HbA1c concentrations and risk of diabetes-related complications in longer trials. Future trials with ORMD-0801 are planned and include a euglycemic clamp with glucose tracer study to improve our understanding of the pharmacodynamic properties and hepatic effect of this preparation, a dose escalation study to elucidate the full acute therapeutic hypoglycemic potential of the drug, and long term trials with a larger cohort of type 2 and type 1 diabetes patients to measure the drug's impact on HbA1c, prolonged glycemic control and application as a novel drug for diabetes. These studies, will shed light on the pharmacodynamic mechanism and full therapeutic potential of this oral insulin preparation in promoting better control of patients with type 1 diabetes.

## Supporting Information

Checklist S1 **Consort/TREND checklist.**
(PDF)

Protocol S1 **Clinical trial protocol.**
(DOC)
