# Supplementary material for: Glucose-Reducing Effect of the ORMD-0801 Oral Insulin Preparation in Patients with Uncontrolled Type 1 Diabetes: A Pilot Study
Source: PLoS One. 2013 Apr 9;8(4):e59524. doi: 10.1371/journal.pone.0059524 (PMC3622027; doi:10.1371/journal.pone.0059524)
Supplement: Protocol S1 — Clinical trial protocol. (DOC) [file pone.0059524.s002.doc]

**Clinical Protocol** – November 2008

The Effectiveness of oral insulin in preventing **hypoglycemia** in people with unstable type 1 diabetes.

**INVESTIGATOR STUDY CENTER**

**Roy Eldor MD (PI)**

Diabetic Unit, 4th Floor

Hadassah University Hospital

Ein Karem, Jerusalem

Israel 91120

**Objective**

- **To determine the safety and tolerability of multi-doses of Orameds oral insulin in people with type 1 diabetes**

Secondary Outcome Measures:

**To determine if oral insulin is likely to improve glycemic stability assessed by the reduction of the frequency and severity of hypoglycemic episodes in people with unstable type 1 diabetes**

**Diabetes** has emerged as a primary health threat of the 21^st^ century. The condition formally termed *diabetes mellitus* is a hormone disorder signified by the cells’ inability to absorb glucose from the blood. This can occur either when there is not enough insulin in the blood, or when the body’s cells fail to respond normally to the insulin that is present. In either case, the negative results are manifold: First, the blood becomes flooded by an overabundance of sugar, sending glucose concentrations in the blood shooting up to dangerous levels. Second, without insulin, muscle and liver cells cannot absorb glucose from the blood, so these cells lack sufficient sugar and are therefore starved for energy. As a result, the body’s supply of fats and proteins must be consumed for “fuel” to keep normal physiologically processes going, simultaneously both depleting the body’s energy storages and disrupting normal metabolic regulation of fats and proteins.

Hypoglycemia is prevalent in diabetes. The DCCT reported a threefold increase in severe hypoglycemia and coma in intensively treated patients as compared to conventionally treated patients. An intensively treated individual with type 1 diabetes can experience up to 10 episodes of symptomatic hypoglycemia per week and severe temporarily disabling hypoglycemia at least once a year. An estimated 2-4% of deaths of people with type 1 diabetes have been attributed to hypoglycemia. Hypoglycemia is less common and generally less severe in type 2 diabetes, but is not uncommon in clinical trials using insulin to achieve good metabolic control.

Hypoglycemia can cause severe physical and/or psychosocial morbidity and sometimes death, depending on its severity or duration. Central nervous system glucose deprivation (neuroglycopenia) is of major concern as it can lead to irreversible brain damage and result in cognitive impairment, seizure disorder and emotional lability. Moreover, the fear and concern of hypoglycemia often precludes tight glycemic control and limits full realization of glycemic control’s long-term benefits in preventing diabetes associated complications in the vast majority of patients. Hypoglycemia is often the cause of autonomic dysfunction and thus the more episodes of hypoglycemia a patient has, the more the body’s glucoregulatory response is blunted, decreasing the patient’s awareness of a pending hypoglycemic episode (hypoglycemia unawareness). The concept of hypoglycemia-associated autonomic failure in diabetes posits that recent antecedent hypoglycemia causes both defective glucose counter regulation and hypoglycemia unawareness so that antecedent hypoglycemia leads to a vicious cycle of recurrent hypoglycemia and further impairment of glucose counter regulation. Put in other words, the more episodes of hypoglycemia a patient has, the more the body’s response is blunted, decreasing the patient’s awareness of an episode. Insulin excess due either to endogenous secretion or to exogenous doses appears to be the most consistent cause of hypoglycemia and iatrogenic hypoglycemia is the most common scenario. However, other factors such as dietary intake, physical activity, alcohol use, and drug interactions also may increase the risk of hypoglycemia. Furthermore, recent studies strongly suggest that abnormalities in glucose counter regulation are important—and perhaps dominant—factors in the development of severe hypoglycemia.

Among insulin dependent diabetic subjects there is a subgroup of individuals who exhibit swings of extremes of hyperglycemia to hypoglycemia for no obvious reason. Such patients are defined as having the syndrome of “unstable” or brittle diabetes and they tend to defy all attempts at orthodox glycemic control and the condition disrupts the lives of themselves, their relatives and their healthcare teams, recurrent and long hospital admissions are the rule. The peak age-group for brittle “unstable” diabetes is 15-30, but it can occur in older patients as well as in younger individuals. Suggested causes included subcutaneous insulin degradation, impaired hyperemic responses to insulin injection, and enhanced insulin clearance. Presently, it is believed that "Pure" recurrent hypoglycemia is associated with loss of awareness of low glucose concentration. The syndrome of recurrent severe and asymptomatic hypoglycemia, associated with delayed adrenergic and autonomic counterregulatory responses, has been recognized in patients receiving intensified insulin treatment and may result from rather than be the initiating cause of recurrent hypoglycemia. The vicious circle that results, however, makes it particularly dangerous for these patients as it is reported to be a cause of mortality amongst them.

The normal counter regulatory responses to hypoglycemia is basically as follows; a decrease in plasma glucose normally triggers a cascade of reactions, mostly hormonal, that rapidly return the glucose concentration to baseline levels. Ultimately there is an increase in glucose production in the liver (and kidneys) and a decrease in peripheral glucose utilization. Both mechanisms act in opposition to the effects of insulin and, hence, result in the reversal of hypoglycemia.

Different counter regulatory mechanisms are activated at different threshold levels of glucose concentration. The first defense against a falling plasma glucose concentration is a decrease in endogenous insulin secretion. This mechanism is critical in patients with residual endogenous insulin secretion. Of the other hormones, epinephrine and glucagon appear to be the most potent counter regulatory factors and are the primary fast-acting hormones in the defense against acute hypoglycemia. These hormones are secreted promptly after plasma glucose levels fall, and both induce a rapid increase in endogenous glucose production. Glucagon acts to increase endogenous glucose production and does so via increases in hepatic glycogenolysis and gluconeogenesis, providing three carbon glucose substrates (lactate, pyruvate, alanine, and glycerol). Epinephrine can also acutely increase endogenous glucose production. Epinephrine has effects similar to glucagon on hepatic glucose production but can also stimulate net renal glucose production. Additionally, epinephrine has an important physiological function in reducing insulin-stimulated glucose uptake. Other major counter regulatory hormones seem to be less critical in the initial phase of a hypoglycemic episode but are important in the later stage of glucose stabilization. Cortisol and growth hormone both help the liver to sustain glucose output in the face of hyperinsulinemia. In addition, these hormones reduce peripheral glucose use during recovery from hypoglycemia, an action shared by epinephrine.

An appropriate increase in endogenous glucose production (EGP) is critical for the recovery from **hypoglycemia. B**oth Glucagon and epinephrine act to increase endogenous glucose production via increases in hepatic glycogenolysis and gluconeogenesis, the two major sources of glucose-6-phosphate destined for dephosphorylation and glucose release. Glycogenesis and glycogenolysis take place simultaneously, with the preponderance favoring glycogenolysis when an increase in EGP is required, whereas gluconeogenesis is favored in the fasting, postabsorptive state. Recent studies suggest that glycogen breakdown may be the primary mechanism responsible for the increase in EGP during the initial recovery from insulin-induced hypoglycemia, followed by an increase in gluconeogenesis.

Type 1 diabetic subjects have an impaired hepatic glycogen metabolism characterized by reduced glycogen content, glycogen being the principal short-term reserve for circulating glucose. Patients with poorly controlled type 1 diabetes accumulate poor hepatic glycogen stores during feeding and consequently they sustain limited rates of endogenous glucose production during fasting. The maintenance of fasting euglycemia in response to a drop in blood glucose levels becomes more dependent on the activation of gluconeogenesis by counter regulatory mechanisms than on glycogen breakdown. For many type 1 diabetic subjects this defect increases their vulnerability to a hypoglycemic episode, particularly fasting hypoglycemia

Glycogen synthesis is regulated by portal vein concentrations of insulin and glucagon. The normalization of hepatic glycogen metabolism in type 1 diabetic patients can only be achieved by increased portal vein insulin (insulin/glucagon ratio) that favors glycogen synthesis which stimulates glycogen synthesis flux, inhibits glycogen phosphorylase flux to result in suppression in the rate of hepatic glycogen cycling and an increase in the rate of net hepatic glycogen synthesis.

A shift in insulin to glucagon ratio (in favor of glycogenesis) in the portal vein can be achieved either by systemic administration of insulin (parenteral administration) or by administering insulin orally (insulin that is ferried directly to the portal vein). Because of the natural gradient 3:1 gradient of portal/peripheral concentration of insulin, administering insulin systemically is likely to require large amounts of insulin which are likely to place the individual at risk for hypoglycemia. In contrast, oral insulin administration restores (albeit temporarily) the high portal insulin levels required for glycogenesis with no systemic hyperinsulinemia (minimal amounts of insulin spills over to the periphery) and is thus devoid of the risk of hypoglycemia.

Oramed Pharmaceuticals inc. has developed an oral insulin dosage form which has undergone extensive preclinical and clinical evaluation and is currently in phase 2 clinical trials. Clinical results up until now have demonstrated that oral insulin administration is safe and well tolerated by healthy individuals as well as by people with type 1 and type 2 diabetes. Orameds’ oral insulin is absorbed from the gastrointestinal tract to result in consistent reduction in blood glucose, reduction in c-peptide and does not cause hypoglycemia.

The intent of this proposed study is to assess the potential of oral insulin to mitigate diabetic instability and reduce the frequency and severity of hypoglycemic episodes in patients with type 1 diabetes. Restoring hepatic glycogen stores is critical for the normalization of conterregulation to hypoglycemia in these patients. Oral insulin can provide a means to increase portal insulin levels, shift the ratio of insulin/glucagon in favor of glycogenesis while sparing the patient the risk of hypoglycemia due to peripheral hyperinsulimia.

**Proposed Mechanism:**

Any attempt to develop an oral insulin modality must take into account two major obstacles that result from insulin’s biochemical character as a polypeptide: 1) Its direct transfer across the mucosal barrier is restricted; 2) it is subject to degradation by the proteolytic enzymes located in the stomach and intestinal lumen.

To overcome these barriers, our proposed mechanism utilizes several co-factors to prevent the digestion of the introduced hormone in the gastrointestinal tract and to facilitate its physiological absorption. After performance of a range of studies for optimization of co-factors, we have identified the most efficacious formulation of encapsulated oral insulin. The proposed composition contains: (1) crystalline insulin, (2) a digestion enhancer, and (3) a formula protector, (4) Omega 3-rich fish oil, in a coated capsule. These components are expanded upon in greater detail below. Each has a target function which promotes the goals of our treatment modality. The enhancer functions as an effective enhancer of the mixture. The protectors prevent enzymatic degradation of the insulin by mucosal enzymes.

.

**Study Design**:

- The patient will be screened and an informed consent be obtained. A total of 8-12 type 1 diabetic patients with known uncontrolled type 1 diabetes will be recruited into this study at one investigational site. Patients will remain on their current medication with no changes to dose or regimen. Patients will undergo 5 days of run-in period whereby data from the CGM will be collected to assess the glycemic swings. The data will serve as a base-line for comparison. After the 5 day run-in period patients will be administered oral insulin for duration of 10 days. Oral insulin will be administered in one capsule given 45 min. before each meal. (The intervals of 5, and 10 days were chosen based on the life of the sensor- The average sensor lif3e is 5 days at which point it needs to be changed).
- Hypoglycemic episodes will be assed by clinical reporting and by Continuous glucose monitoring (CGMS). CGMS consists of a sterile disposable subcutaneous glucose-sensing device connected by a cable to a glucose monitor. The system allows downloading and reviewing of the data on a personal computer. The sensing device (needle) detects glucose levels in the subcutaneous interstitial fluid electrochemically (reaction with glucose oxydase) and registers it at frequent intervals, the data collected is stored and will be analyzed at the completion of the study.
- Hypoglycemia severity grading: Mild (grade 1) - Patient is aware of, responds to and self-treats the hypoglycemia Moderate (grade 2) -Patient cannot respond to hypoglycemia and requires help from someone else, but oral treatment is successful, Severe (grade 3) - Patient is semi-conscious or unconscious or in coma ± convulsions and may require parenteral therapy (glucagon or IV glucose)

**STUDY DURATION**:

**Screening Phase:** up to 14 days prior to first dose of study medication

Pre treatment phase: 5 days

**Treatment Phase:** 10 days.

**End of Study:** After completion of Period 2 or at early discontinuation

**SCREENING PHASE**:

Screening may take place up to 14 days prior to first dose of study drug administration. The following evaluations will be performed after the subject signs informed consent:

- Medical history
- Physical examination
- Medication history
- Vital signs (blood pressure, heart rate). Vital signs will be measured in the sitting position after at least 5 minutes of rest.

Clinical laboratory evaluations (chemistry, hematology)

**TREATMENT PHASE**

The Treatment Phase will consist of two periods:

1. Pretreatment phase in which the volunteers will have only his CGM for 5 days.

2. Treatment phase in which the volunteers will start medication before each meal (3 times/day) for 10 days including CGM.

**END OF STUDY/EARLY DISCONTINUATION:**

After completion of Period 2, subjects will complete the following end of study evaluations:

- Vital signs (blood pressure, heart rate) will be measured in the sitting position after at least 5 minutes of rest.
- Clinical laboratory evaluations (hematology, chemistry)
- Physical Exam

Subjects who are discontinued early from the study will complete the end of study evaluations at the time of early discontinuation.

**TREATMENT REGIMEN**:

The treatment regimen is as follows:

1. Period 1: no added medication. 5 days CGM. No change in current treatment.
2. Period 2: For 10 days one capsule of insulin before each meal (3 time/day). Each capsule contains: 8 mg insulin.

**SAFETY EVALUATIONS:**

- Adverse events will be collected throughout the study beginning from the time the subject signs the consent form until the end of study evaluations
- Concomitant medications/therapies will be recorded throughout the duration of study, beginning from the time the subject signs the informed consent.
- Vital signs (blood pressure, heart rate) will be measured in the sitting position after at least 5 minutes of rest at the following times:
- Screening
- At the end of period 1
- End of study
- Clinical laboratory evaluations (chemistry, hematology) will be performed at screening and at end of study or early discontinuation. .
- Physical examinations at screening and end of study or early discontinuation.

**SAMPLE SIZE:**

12-8 Type 1 subject – HbA1C: 7.5-10.

Inclusion Criteria:

- Type 1 diabetes mellitus based on clinical dependence on insulin and positive GAD measurements.
- Male or female aged 18-50 years inclusive
- Body weight between 50 - 100kg and body mass index between 18 and 30 kg/m2
- Diagnosed 6 or more months ago
- No other known medical problems that may affect the safety of the patient or the results of the study (as assessed by the study physician).

Exclusion Criteria:

- History or presence of clinical significant gastrointestinal pathology or symptoms, liver or kidney disease or any condition that might interfere with the absorption, distribution, metabolism or excretion of the drug.
- Positive pregnancy test or lactation
- Lack of family support (patient lives alone)
- Unwilling or unable to follow the study protocol
- Concurrent medical problems

**CONCLUSION**:

Diabetes today affects more than 200 million individuals worldwide, a number expected to double over the next 25 years. The majority of these patients suffer from Type II diabetes, which now strikes an increasing number of children and young people, as well as adults of middle age and the elderly. In current practice, Type I patients receive insulin while diet and oral hypoglycemic agents are used to treat Type II sufferers. However, this mode of treatment fails to provide sufficient glycemic control in 30-40% of Type II cases. Hence, there is an increasing trend is to treat Type II patients as well with insulin, administered either as monotherapy or in combination with the oral hypoglycemic drugs. With properly administered insulin therapy, improvements of metabolic control can be attained and further deterioration of pancreatic function is prevented. However, the current subcutaneous insulin regimen is ideal neither for patient quality of life nor for physiological efficacy. Treatment with oral insulin, on the other hand, can provide an enhanced physiological effect by mimicking the natural passage of endogenous insulin through the liver. In addition, the pain-free and comfortable administration of insulin will increase patients’ compliance to therapy, which, in turn, will decrease the long-term complications of diabetes. However, several obstacles have stood in the way of the development of an oral insulin formulation, primarily the need to prevent degradation of the ingested hormone by proteases and to enable its absorption across mucosal membranes.

Our studies have confirmed the feasibility of lowering blood glucose levels of Type II diabetics with an orally administrated formulation of insulin that is both safe and effective. Specifically, our novel form of encapsulated insulin, containing the necessary protecting and promoting ingredients (enhancers and antiproteases), has led to observed decreases of plasma glucose of up to 35%.
